# Validation of commonly used reference genes for sleep-related gene expression studies

**DOI:** 10.1186/1471-2199-10-45

**Published:** 2009-05-15

**Authors:** Kil S Lee, Tathiana A Alvarenga, Camila Guindalini, Monica L Andersen, Rosa MRPS Castro, Sergio Tufik

**Affiliations:** 1 Associação Fundo de Incentivo à Psicofarmacologia (AFIP), Rua Napoleão de Barros, 925, Vila Clementino, São Paulo 04024-002, SP, Brazil; 2 Department of Psychobiology, Universidade Federal de São Paulo (UNIFESP), Rua Napoleão de Barros, 925, Vila Clementino, São Paulo 04024-002, SP, Brazil

## Abstract

**Background:**

Sleep is a restorative process and is essential for maintenance of mental and physical health. In an attempt to understand the complexity of sleep, multidisciplinary strategies, including genetic approaches, have been applied to sleep research. Although quantitative real time PCR has been used in previous sleep-related gene expression studies, proper validation of reference genes is currently lacking. Thus, we examined the effect of total or paradoxical sleep deprivation (TSD or PSD) on the expression stability of the following frequently used reference genes in brain and blood: *beta-actin (b-actin), beta-2-microglobulin (B2M), glyceraldehyde-3-phosphate dehydrogenase (GAPDH)*, and *hypoxanthine guanine phosphoribosyl transferase (HPRT)*.

**Results:**

Neither TSD nor PSD affected the expression stability of all tested genes in both tissues indicating that *b-actin, B2M, GAPDH *and *HPRT *are appropriate reference genes for the sleep-related gene expression studies. In order to further verify these results, the relative expression of *brain derived neurotrophic factor (BDNF) *and *glycerol-3-phosphate dehydrogenase1 (GPD1) *was evaluated in brain and blood, respectively. The normalization with each of four reference genes produced similar pattern of expression in control and sleep deprived rats, but subtle differences in the magnitude of expression fold change were observed which might affect the statistical significance.

**Conclusion:**

This study demonstrated that sleep deprivation does not alter the expression stability of commonly used reference genes in brain and blood. Nonetheless, the use of multiple reference genes in quantitative RT-PCR is required for the accurate results.

## Background

Sleep is a complex phenotype that involves several neurochemical and physiological processes. It is known to perform restorative functions and to facilitate memory consolidation [1,2]. Although sleep is essential for overall well-being and optimal physical and psychological functioning, chronic sleep restriction is frequently experienced due to contemporary social and domestic responsibilities, medical conditions and sleep disorders [3]. Sleep restriction alters sleep architecture primarily by decreasing the duration of the REM (rapid eye movement) sleep stage, also known as paradoxical sleep (PS) [3]. Therefore, approaches that reduce or abolish PS have been used to simulate chronic sleep restriction.

Persistent sleep debt impairs neurobehavioral functions [3,4] and increases the risk for chronic diseases such as cardiovascular disorders [5], erectile dysfunction [6] and diabetes [7]. Thus, careful characterization of the effects of sleep deprivation not only improves our knowledge about the complex sleep process but also contributes to elucidation of the mechanism underlying these chronic diseases and their treatment. Remarkably, sleep genetics has been emerging as one of the important features in sleep research [8,9]. Several studies have demonstrated that genetic background or certain gene products can affect individual sleep patterns [10]. Investigation of these candidate genes can help identify the molecular machinery responsible for both normal sleep and sleep-related disorders.

Reverse transcription (RT) followed by quantitative real time PCR (qPCR) is one of the most compelling approaches for gene expression analysis due to speed and simplicity of the method [11]. Potential methodological variations can be corrected by normalizing the gene expression of interest to a set of references, frequently referred to as housekeeping genes, which presumably maintain constitutive expression. However, increasing evidence has demonstrated that the expression of commonly used reference genes, such as *b-actin *and *glyceraldehyde-3-phosphate dehydrogenase (GAPDH)*, can vary under certain circumstances [12,13]. Consequently, the selection and validation of reference genes for the tissue and experimental conditions of interest is a critical step to generate reliable results using RTqPCR methodology.

Herein, the expression stability (M) of four common reference genes (Table 1) was evaluated in brain and blood collected from control and sleep deprived rats, using GeNorm software [14]. In order to validate the selected reference genes, the expression of *brain derived neurotrophic factor *(*BDNF*) and *glycerol-3-phosphate dehydrogenase1 *(*GPD1*) was examined in brain and blood, respectively, as the expression of these genes has been shown to vary with sleep deprivation [15,16].

**Table 1:** Candidate genes with respective gene accession number and primer sequences

Gene name	Access number	Primer sequences (5'-3')
beta-actin	NM_031144	AGCGTGGCTACAGCTTCACC
		AAGTCTAGGGCAACATAGCACAGC
beta-2-microglobulin (B2M)	Y00441	GCCATCCACCGGAGAATG
		GGTGGAACTGAGACACGTAGCA
glyceraldehyde-3-phosphate	NM_017008	TGCCCCCATGTTTGTGATG
dehydrogenase (GAPDH)		GCTGACAATCTTGAGGGAGTTGT
hypoxanthine guanine	NM_012583	GCGAAAGTGGAAAAGCCAAGT
phosphoribosyl transferase (HPRT)		GCCACATCAACAGGACTCTTGTAG
brain derived neurotrophic	NM_012513	ATGCCGAACTACCCAATCGT
factor (BDNF)		GCCAATTCTCTTTTTGCTATCCA
glycerol-3-phosphate	NM_022215	TGGCCCCTTTCCAAGGTT
dehydrogenase 1 (GPD1)		TCCAGGCTGCTGATCTGTGA

## Results

### RNA quality

**Figure 1:**
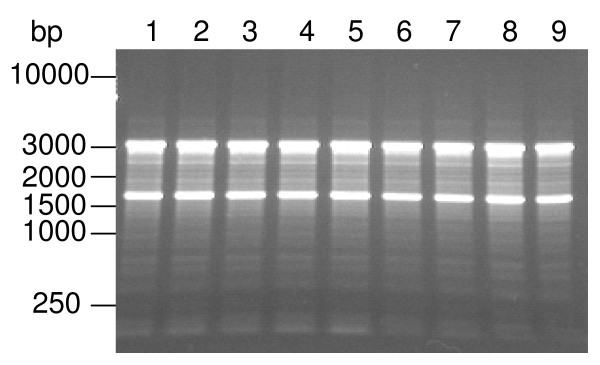
**Representative image of electrophoresis of total RNA in agarose gel**. Total RNA extracted from brain of controls (1, 2, 3) and rats subjected to paradoxical sleep deprivation without (4, 5, 6) or with sleep recovery (7, 8, 9) was fractioned in agarose gel 1%. Approximately 1.5 μg of RNA was loaded for each sample. Intact 28 S and 18 S rRNA were observed without higher molecular weight molecules.

RNA quality is one of the most important factors that determine the precision of RTqPCR. In order to evaluate the quality of the DNase I treated RNA, we performed electrophoresis in agarose gel. All RNA samples used in this study exhibited intact 28 S and 18 S rRNA and similar banding patterns (Figure 1), indicating that degradation of RNA was negligible. Also, the absence of high molecular weight molecules suggested that contamination with genomic DNA was minimal. These data demonstrated that the RNA samples used for this study were of appropriate quality to perform RTqPCR.

### RTqPCR

Pilot experiments were performed with *b-actin *primers to determine the optimal amounts of RNA and cDNA that result in Ct values within the linear range. A total of 0.3 μg of RNA, treated with DNase I, was used for the 20 μL reverse transcription reaction and 2 μL of cDNA was used for qPCR. Subsequently, the same amounts of RNA and cDNA were used for all reactions producing Ct values within 15.0 to 33.0 ranges. Analysis of each amplification product produced a dissociation curve containing a single peak with narrow melting temperature (Table 2), indicating that each primer pair amplified a single predominant product.

**Table 2:** Melting temperature (°C) of amplification products with standard deviations.

Tissue	Beta-actin	B2M	GAPDH	HPRT	GOI
Brain	81.0 ± 0.2	79.5 ± 0.2	77.7 ± 0.3	78.2 ± 0.2	77.9 ± 0.1
Blood	81.1 ± 0.2	79.6 ± 0.2	77.7 ± 0.3	78.3 ± 0.2	80.1 ± 0.3

GOI: gene of interest (*BDNF *for brain and *GPD1 *for blood).

### Expression stability of the reference genes

The expression of *b-actin, B2M, GAPDH *and *HPRT *was measured in two independent experiments where the rats were distributed into three groups: controls (C), animals subjected to paradoxical sleep deprivation for 96 hours (PSD96) and animals subjected to PSD96 and then a sleep recovery period of 24 hours (SR24). In the second experiment, a group of animals subjected to total sleep deprivation for 6 hours (TSD6) was included. For all samples, qPCR was performed at least in duplicate and reproducible Ct values were obtained with correlation coefficient > 0.99 (p < 0.0001) for both experiments.

The data from C, PSD96 and SR24 groups were arrayed in a single matrix for the calculation of M values. To assess the variability between the experiments, M values were calculated for each experiment separately. The data of TSD6 and respective control groups were analyzed in a distinct matrix in order to discriminate the effect of PSD from the TSD. As shown in Table 3, all genes presented relatively low M values for both protocols of sleep deprivation. Although the best pair of reference genes (lowest M values) varied between the two experiments, the four genes can be considered suitable reference genes for sleep-related gene expression studies, according to the previously suggested cut-off of M value (0.5) for a stable reference gene [17].

**Table 3:** Reference genes with their respective M-values in three distinct experiments.

	*Brain*			*Blood*		
	*Exp_1*	*Exp_2*	*Exp_3*	*Exp_1*	*Exp_2*	*Exp_3*
Actin	0.163	0.160	0.152	0.234	0.159	0.217
B2M	0.110	0.160	0.166	0.309	0.226	0.162
GAPDH	0.092	0.195	0.197	0.157	0.159	0.162
HPRT	0.092	0.176	0.152	0.157	0.370	0.377

Exp_1 and Exp_2 contained 3 groups: Control, PSD96 and SR24. Exp_3 contained 2 groups: control and TSD6.

To distinguish the effect of sleep deprivation on gene expression stability, M values were also calculated for each group (C, PSD96, SR24 and TSD6) independently, but all M values were below the cut-off (0.5), suggesting that total or paradoxical sleep deprivation did not alter the expression stability of commonly used reference genes.

### Validation of the reference genes

To validate the selected reference genes, the expression of *BDNF *was determined by RTqPCR in brain. Relative expression fold changes were calculated by the 2^-ΔΔCT ^method [18] using *b-actin, B2M, GAPDH *or *HPRT *as the reference genes as well as by using the normalization factor (NF) derived from the geometric mean of four reference genes [14].

**Figure 2:**
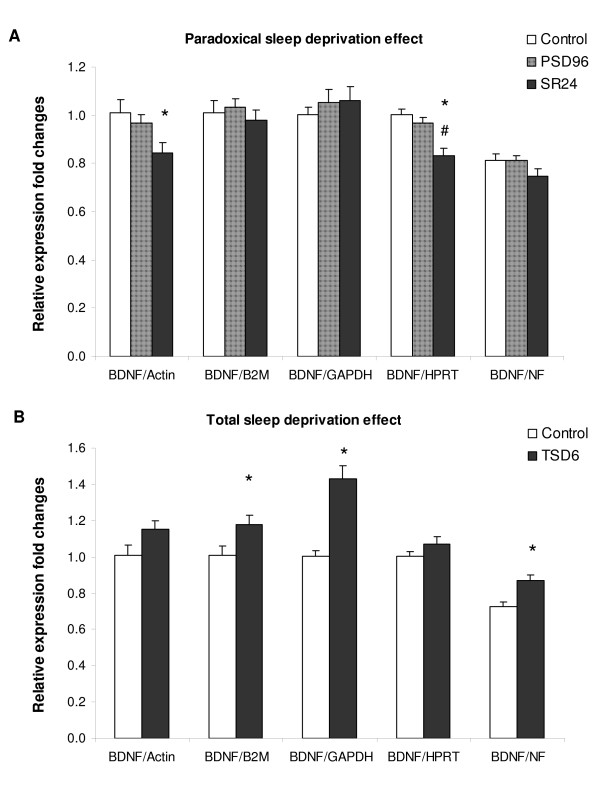
**Expression of *BDNF *in the brain**. **A**. The expression of *BDNF *was quantified by RTqPCR in the brain of controls (C), animals subjected to paradoxical sleep deprivation for 96 hours (PSD96), and animals subjected to PSD and sleep recovery for 24 hours (SR24). **B**. The expression of *BDNF *was also evaluated in animals subjected to total sleep deprivation for 6 hours (TSD6) and compared to the control (C). The relative expression fold changes were calculated using the 2^-ddCt ^method for each reference gene or the normalization factor (NF) generated by GeNorm software. Error bar represents standard error of the mean. * p < 0.05 compared to control, # p < 0.05 compared to PSD (ANOVA followed by Tukey's post hoc test).

The normalization with each reference gene or with the NF produced consistent results showing that PSD96 did not alter the *BDNF *expression compared to the control (Figure 2A, p > 0.05). However, conflicting results were obtained regarding the effect of sleep recovery. While *b-actin *or *HPRT *generated slight, but statistically significant decrease in *BDNF *expression for the SR24 group (p < 0.05 and p < 0.001 for *b-actin *and *HPRT *respectively), the use of *B2M, GAPDH *or NF as references did not produced a similar reduction compared to the control (p > 0.05, Figure 2A).

In a similar way, the effect of TSD6 was also statistically variable depending on the selection of the reference gene: normalization with *B2M, GAPDH *or NF resulted in significant increase of *BDNF *expression compared to the control (p < 0.05), while the use of *b-actin *or *HPRT *did not validate the effect of TSD (p > 0.05, Figure 2B).

**Figure 3:**
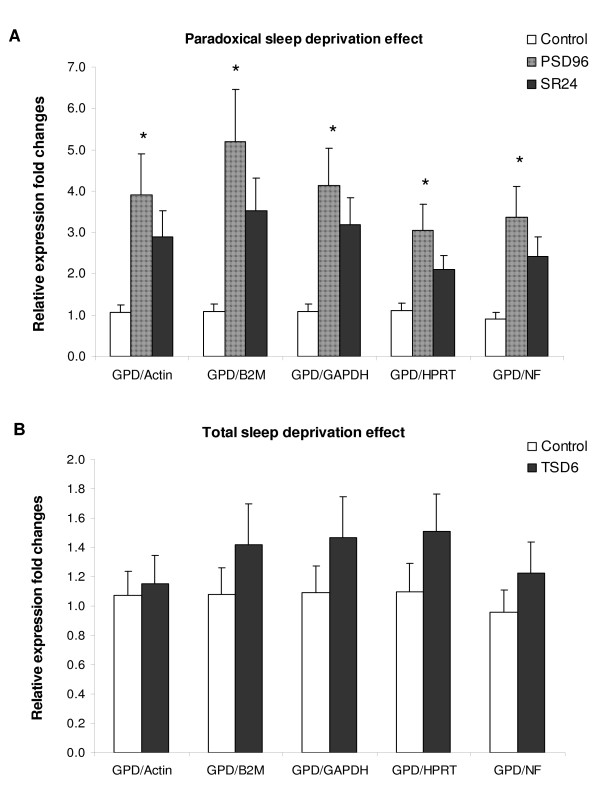
**Expression of *GPD1 *in the blood**. **A**. The expression of *GPD1 *was measured by RTqPCR in the blood of controls (C), animals subjected to paradoxical sleep deprivation for 96 hours (PSD96) and animals subjected to PSD and sleep recovery for 24 hours (SR24). **B**. The expression of *GPD1 *was also evaluated in animals subjected to total sleep deprivation for 6 hours (TSD6) and compared to the control (C). The relative expression fold changes were calculated using the 2^-ddCt ^method for each reference genes or the normalization factor (NF) generated by GeNorm software. Error bar represents standard error of the mean. * p < 0.05 compared to control (ANOVA followed by Tukey's post hoc test).

In blood, the expression of *GPD1 *was evaluated since sleep and metabolism are closely related and that *GPD1 *is one of the key intermediates between the carbohydrate and lipid metabolism [19]. All reference genes and NF resulted in a significant increase of *GPD1 *expression in PSD96 group compared to the controls (p < 0.05) but the magnitude of fold changes was somewhat different between each analysis (Figure 3A). In SR24 group, the mean relative expression was increased by at least two fold compared to the controls, but it did not reach statistical significance, probably due to high individual variability (p > 0.05).

TSD6 was not sufficient to produce similar increase in *GPD1 *expression, and all reference genes and NF generated consistent results (p > 0.05, Figure 3B).

## Discussion

### Effects of sleep deprivation on the expression of classical reference genes

Given the importance of the genetic aspect of sleep research, substantial efforts were made to identify genes involved in sleep-wake cycles. These studies reported that sleep deprivation can modify the expression of genes frequently used as internal controls for RTqPCR [20]. For instance, short-term sleep deprivation altered the expression of cytoskeletal proteins such as beta-actin and tubulin in two independent proteomic analyses [12,21]. Furthermore, Biswas and colleagues showed that the amount of actin and tubulin decreased in the brains of paradoxical sleep-deprived rats and culminated in neuronal apoptosis [22]. *GAPDH *is another classical reference gene that requires careful analysis before using in sleep-related gene expression studies. Several studies demonstrated that sleep deprivation or sleep disorders alter glucose metabolism and enhance the risk for type 2 diabetes [7]. Thus, despite lack of direct evidence, the strong correlation between sleep and glucose metabolism suggests that the expression and/or activity of *GAPDH *might be modulated by the sleep-wake cycle.

These findings strongly justified the importance of the present study and our data demonstrated that the most commonly used reference genes presented stable expression throughout the total or paradoxical sleep deprivation period, as the M values were below the cut-off previously suggested for stable genes [17].

### Expression of BDNF in the brain

Brain-derived neurotrophic factor (BDNF) is an important mediator of memory and cognition and its expression is strongly modulated by neuronal activity [15]. In our previous study, we observed that the PSD96 increased the expression of *BDNF *in cortical tissue [16] while other group showed that *BDNF *expression was reduced by 6 h of PSD in the cerebellum and brainstem [23]. In the present study, we did not observe any alteration of *BDNF *expression in paradoxical sleep deprived rats. Conceivably, distinct regulation of *BDNF *expression may occur throughout the different regions of the brain and therefore, the use of whole brain might mask the effect of PSD on specific region of the brain. On the other hand, the intracerebral injection of exogenous BDNF in rat increased the time spent in NREM sleep without affecting REM sleep [24]. These data spare the possibility of *BDNF *expression being minimally affected by PSD in many areas of the brain corroborating the present findings.

In contrast, the short-term total sleep deprivation appears to up-regulate the expression of *BDNF *in various regions of the brain [15,25-27] suggesting that the use of whole brain would not interfere with the gene expression results. As expected, an overall increase in mean relative expression of *BDNF *was observed in TSD6 group, but the statistical significance was achieved only when *B2M*, *GAPDH *or NF were used for the normalization, although all tested reference genes presented stable expression stability. These data illustrated that use of single reference gene can produce flawed results leading to misinterpretation of physiological events. Thus, the application of multiple reference genes in RTqPCR is desirable for reliable results.

### Expression of GPD1 in blood

The reduction of sleep time and augment of obese individuals in modern life are concurrent trends indicating that sleep-wake cycle has strong impacts on the energy metabolism [28]. GPD1 is a NAD+ dependent cytosolic enzyme that generates key intermediates between glucose and lipid metabolism [19]. In our previous studies, we found that *GPD1 *expression was increased in cortex and blood of rat subjected to PSD96 compared to controls [16]. Although the increase of *GPD1 *expression followed by PSD96 was confirmed in this study, short-term TSD did not have significant effect on the expression of this gene. Several studies have demonstrated that the alteration of biochemical parameters can occur even after short period of sleep deprivation [29-31]. Thus, the increase of *GPD1 *expression occurred after PSD96 can be a secondary effect of prolonged paradoxical sleep loss. Finally, the fact that both *BDNF *and *GPD1 *expression was differently modulated by PSD and TSD demonstrates the importance of distinct protocols of sleep deprivation in order to discriminate events that happen in each stage of sleep at distinct time course.

## Conclusion

This study demonstrated that the most commonly used reference genes are suitable for gene expression studies that involve sleep deprivation. However, more reliable results of RTqPCR can be obtained when multiple reference genes are used.

## Methods

### Selection of reference genes

In order to avoid possible co-regulation of expression, genes belonging to distinct biological pathways were selected as follows: *beta-actin (b-actin), glyceraldehyde-3-phosphate dehydrogenase (GAPDH), beta-2-microglobulin (B2M) *and *hypoxanthine guanine phosphoribosyl transferase (HPRT)*. *Brain derived neurotrophic factor (BDNF) *and *glycerol-3-phosphate dehydrogenase 1 (GPD1) *were used to validate the reference genes. Gene accession numbers as well as primer sequences are listed in Table 1.

### Sleep deprivation

Adult male Wistar-Hannover rats were assigned to three groups (3 animals/group): home-cage controls (C), rats subjected to PSD for 96 hours (PSD96) [32], and rats subjected to PSD96 with an additional sleep recovery period of 24 hours (SR24). In the second experiment, 9 animals per group were used and an additional group of 8 animals was included for a total sleep deprivation of 6 hours (TSD6). During the PSD, the rats were placed on narrow circular platforms located inside a tank (143 cm × 41 cm × 30 cm), which is filled with water of ~1 cm depth. When rats reach the paradoxical phase of sleep, they fall into the water, due to muscle atonia, and wake up. This protocol causes deprivation of all sleep stages on the first day, and then becomes more selective leading to the complete loss of paradoxical sleep on subsequent days. TSD was achieved by gentle handling method as described elsewhere [33]. The rats used in this study were maintained and treated according to the ethical and practical guidelines for the use of laboratory animal. The experimental protocol has the approval of the Ethical Committee of UNIFESP (CEP N. 05/434). Maximum efforts were taken to use the smallest, but enough number of animals in the experiments to ensure unambiguous and reliable statistical analysis and data interpretation.

### Tissue collection and total RNA extraction

The animals were decapitated immediately after sleep deprivation and sleep recovery procedure. The brain was rapidly dissected, flash frozen in liquid nitrogen, and then stored at -80°C until RNA extraction. Total RNA was extracted from whole tissues using Trizol reagent (Invitrogen) according to the manufacturer's instructions. After decapitation, neck blood samples (2.5 mL) were collected in PaxGene RNA collection tubes (PreAnalytiX) according to the manufacturer's recommendations. Two hours after collection, total RNA was extracted using PaxGene blood RNA isolation kit (PreAnalytiX) with minor modification [34]. After RNA extraction, RNA was treated with DNaseI and the quality of the RNA was evaluated by electrophoresis in agarose gel.

### Reverse transcription and quantitative Real time PCR (RTqPCR)

Total RNA was reverse transcribed into cDNA using SuperScript™ III Platinum^® ^Two-Step qRT-PCR kit with SYBER^® ^Green (Invitrogen). Reverse transcription was performed at 25°C for 10 min, 42°C for 50 min and then 85°C for 10 min. Each cDNA sample was then used as a template for real-time PCR amplification using the same kit (Invitrogen). Amplification and detection was performed using an Applied Biosystems 7500 Real-Time PCR system (Applied Biosystems) according to the manufacturer's instructions using a two-stage cycle (95°C for 15 s and 60°C for 1 min) repeated 40 times followed by a dissociation stage.

### Data analysis

Gene stability was evaluated using GeNorm algorithm, freely available for download . The GeNorm algorithm relies on the principle that the expression ratio of two ideal reference genes must be constant between samples [14]. This software calculates the variation of this ratio for all two-by-two combinations of reference genes. Lower M values indicate higher expression stability, with 0.5 being a suggested cut-off for stable genes [17]. The expression fold changes of *BDNF *and *GPD1 *were calculated by the 2^-ΔΔCT ^method for each reference genes [18], or by the 2^-ΔCT ^method using the geometric mean of suitable reference genes as the normalization factor [14]. Unpaired t-test or Analysis of variance (ANOVA) followed by Tukey post hoc test was conducted on the relative expression values using software GraphPad Prism (v 4.00).

## List of abbreviations

*(b-actin)*: *Beta-actin*; *(B2M)*: *beta-2-microglobulin*; *(BDNF)*: *brain derived neurotrophic factor*; (C): Control group; (Ct): threshold cycle; *(GAPDH): glyceraldehyde-3-phosphate dehydrogenase; (GPD1): glycerol-3-phosphate dehydrogenase 1; (HPRT): hypoxanthine guanine phosphoribosyl transferase*; (PSD96): paradoxical sleep deprivation for 96 hours; (RTqPCR): reverse transcription quantitative polymerase chain reaction; (SR24): sleep recovery for 24 hours; (TSD6): total sleep deprivation for 6 hours.

## Authors' contributions

KSL performed all RTqPCR experiments and was the primary author of the manuscript. TAA performed the sleep deprivation procedure and brain tissue collection. CG extracted blood RNA and performed the data analysis. MLA coordinated paradoxical sleep deprivation procedures. RMRPSC extracted brain RNA and participated in study design. ST conceived the study and participated in the study coordination. All authors read and approved the final manuscript.
